# Translation, cross-cultural adaptation, and validation of health and self-management in diabetes questionnaire (HASMID-10) into Brazilian Portuguese

**DOI:** 10.1590/1516-3180.2022.0681.R1.10042023

**Published:** 2023-06-19

**Authors:** Aldair Darlan Santos-de-Araújo, Almir Vieira Dibai-Filho, André Pontes-Silva, Adriana Sousa Rêgo, Dalyla Lima dos Santos, Abraão Albino Mendes, Fábio Henrique Ferreira Pereira, Solange Negreiros de Almeida Bacelar, Bárbara Emanoele Costa Oliveira, Rudys Rodolfo de Jesus Tavarez, Daniela Bassi-Dibai

**Affiliations:** I Physiotherapist and Doctoral Student, Cardiopulmonary Physical Therapy Laboratory, Universidade Federal de São Carlos (UFSCar), São Carlos (SP), Brazil.; II PhD. Physiotherapist, Postgraduate Program in Adult Health Universidade Federal do Maranhão (UFMA), São Luís (MA), Brazil.; III MSc. Bachelor of Physical Education, Postgraduate Program in Physical Therapy, Universidade Federal de São Carlos (UFSCar), São Carlos (SP), Brazil.; IV PhD. Physiotherapist, Postgraduate Program in Management in Health Programs and Services, Postgraduate Program in Environment, Universidade Ceuma (UNICEUMA), São Luís (MA), Brazil.; V Physiotherapist, Department of Physical Therapy, Universidade Ceuma (UNICEUMA), São Luís (MA), Brazil.; VI Nurse, Postgraduate Program in Management of Health Programs and Services, Universidade Ceuma (UNICEUMA), São Luís (MA), Brazil.; VII Physiotherapist, Postgraduate Program in Environment, Universidade Ceuma (UNICEUMA), São Luís (MA), Brazil.; VIII MSc. Physiotherapist, Postgraduate Program in Dentistry, Universidade Ceuma (UNICEUMA), São Luís (MA), Brazil.; IX PhD. Dentist, Postgraduate Program in Dentistry, Universidade Ceuma (UNICEUMA), São Luís (MA), Brazil.; X PhD. Dentist, Postgraduate Program in Dentistry, Universidade Ceuma (UNICEUMA), São Luís (MA), Brazil; XI PhD. Physiotherapist, Postgraduate Programs in Management in Health Programs and Services, in Environment, and in Dentistry, Universidade Ceuma (UNICEUMA), São Luís (MA), Brazil.

**Keywords:** Quality of life, Diabetes complications, Self care, Surveys and questionnaires, Measurement properties, Psychometric properties, COSMIN tools

## Abstract

**BACKGROUND:**

Considering the ability of the health and self-management in diabetes questionnaire (HASMID-10) to verify the impact of self-management on diabetes, we highlight its relevance to scientific research and clinical applicability. However, to date, no study has been conducted to scientifically support its use in other languages.

**OBJECTIVE:**

To translate, cross-culturally adapt, and validate the HASMID-10 into the Brazilian Portuguese.

**DESIGN AND SETTING:**

A translation, cross-cultural adaptation, and validation study conducted at Ceuma University.

**METHODS:**

Study was conducted in accordance with the Guidelines for the Process of Cross-Cultural Adaptation of Self-Report Measures and Consensus-based Standards for the Selection of Health Measurement Instruments. We included participants of both sexes diagnosed with diabetes, aged between 18 and 64 years, and without cognitive deficits or any other limitations that would prevent them from answering the questionnaire. We assessed participants using the problem areas in diabetes (PAID) scale and HASMID-10. We assessed reliability using a test-retest model with a 7-day interval between assessments. We used intraclass correlation coefficient (ICC), 95% confidence interval (CI), standard error of measurement (SEM), minimum detectable difference (MDD), Spearman correlation coefficient, and floor and ceiling effects.

**RESULTS:**

Sample comprised 116 participants, most of whom were women, overweight, non-practitioners of physical activity, and nonsmokers. We observed significant correlations (P = 0.006; rho = −0.256) between the HASMID-10 and PAID, adequate reliability (ICC = 0.780) and internal consistency (Cronbach's alpha = 0.796). No ceiling or floor effects were observed.

**CONCLUSION:**

HASMID-10 has adequate measurement properties and may be used for Brazilians.

## INTRODUCTION

The diabetes mellitus epidemic has reached an alarming level. It is estimated that by 2030, the disease will affect approximately 578 million individuals, placing it within a problematic public health scenario of an emergency nature and generating great socioeconomic impacts.^1,2^ Currently, Brazil occupies the fourth position in the international ranking of individuals surviving this pathology, reflecting a progressive increase in the number of confirmed diagnoses, especially in the last three decades.^3,4^

Lifestyle changes added to an optimized pharmacological therapy and adherence to physical exercise have been the cornerstone for maintaining glycemic control and consequent improvement in quality of life, reducing complications triggered by the pathology and related diseases, traditionally supported by a team of health professionals who play a significant role in providing guidance on the importance of drugs, food intake, and the benefits of physical activity, whose main objective is to increase adherence to treatment.^5–7^ Despite these efforts, successful adherence to therapy is not always achieved, and this may be related to traditional management approaches where patients are passive recipients of care.^8^

Within this context, self-management can play a significant role in reducing pathology-related complications in the short and long terms. In this sense, self-reported questionnaires have been gaining increasing attention within the self-care scenario because of the ease of administration, time optimization, and active participation of the individual in the care process, in addition to the fact that they are less costly and do not require a specialized team for application.^5,8,9^

In terms of knowledge, the health and self-management in diabetes questionnaire (HASMID-10) was developed to measure the impact of self-management in type 1 and 2 diabetes. The original version has eight items that consider aspects of quality of life and self-management.^10^ However, after psychometric analysis to assess the performance of the questionnaire, two items that provide more details on how emotions and daily activities are affected were inserted, making the most current version composed of 10 questions (HASMID-10) with good psychometric performance and discriminative validity between diabetes types.^11^

Considering the questionnaire's ability to verify the impact of self-management on diabetes, we highlight its relevance to scientific research and clinical applicability. However, to date, no study has been developed to scientifically support its use in other languages.

## OBJECTIVE

Thus, considering that Brazil is one of the countries with the highest number of people living with diabetes, we aimed to translate, cross-culturally adapt, and validate the HASMID-10 into Brazilian Portuguese.

## METHODS

### Study design and ethics aspects

A translation, cross-cultural adaptation, and validation study was conducted in accordance with the Guidelines for the Process of Cross-Cultural Adaptation of Self-Report Measures^12^ and the Consensus-based Standards for the Selection of Health Measurement Instruments (COSMIN).^13^ Authorization to perform the cross-cultural adaptation of the HASMID-10 into Brazilian Portuguese was granted via Oxford University Innovation (https://innovation.ox.ac.uk/outcome-measures/hasmid-10/).

This study was approved by the Research Ethics Committee of Ceuma University in São Luis, Maranhão, Brazil, on August 29, 2018 (number 2.853.570). The participants were recruited through social media, text messaging, and email. All the recruited volunteers provided consent to participate in the study. Data were collected face-to-face in health units in the university community of the city of São Luís (Maranhão, northeastern Brazil) and a community associated with this city, as well as through the online platform Google Forms (Mountain View, California, United States).

### Participants

We based our sampling on the most current and best international guidelines (COSMIN),^13^ and a minimum of 100 participants were recommended. The eligibility criteria were as follows: participants of both sexes, diagnosed with type 1 or type 2 diabetes mellitus, aged > 18 and < 64 years, and without cognitive deficits or any other limitations that would prevent them from answering the questionnaire.

### Translation and cross-cultural adaptation

**Figure 1:**
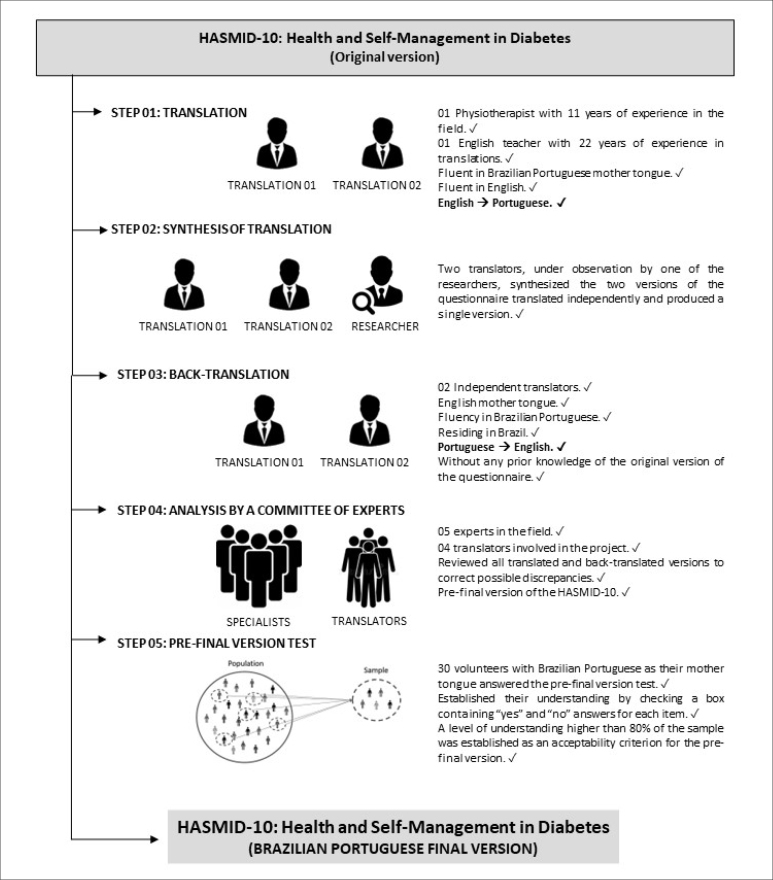
Translation and cross-cultural adaptation process of the HASMID-10 into Brazilian Portuguese.

The translation and cross-cultural adaptation of the HASMID-10 into Brazilian Portuguese followed the criteria shown in Figure 1.

### HASMID-10 Questionnaire

The original version of HASMID^10^ comprises eight attributes, four on quality of life and four on self-care, consisting of eight items with four response options (never, sometimes, usually, and always). Response options were scored from 1 to 4, with a higher score indicating better health-related quality of life and a lower score indicating worse health-related quality of life. The HASMID-10 consists of ten items that cover temper, irritability, hypoglycemic episodes, tiredness, tied to mealtimes, social activities, control, hassle, stress, and support (the original version of HASMID items plus irritability and social activities).^11^ The response options for the HASMID-10 were those of the original (i.e., never, sometimes, usually, and always). The overall questionnaire is scored in reverse and summative, with response levels scored as never = 3, sometimes = 2, usually = 1, and always = 0. Scores range from 0 to 30, with a higher score indicating a better quality of life.

### Problem areas in diabetes

We applied the problem areas in diabetes (PAID) scale, adapted and validated for use in Brazilian Portuguese, to verify construct validity. PAID scale^14^ has 20 questions that range from emotional states frequently reported by patients with type 1 and type 2 diabetes. It also includes questions about aspects of quality of life and emotional problems related to living with diabetes and its treatment, including guilt, anger, depression, worry, and fear. It produces a total score that ranges from 0-100: with a higher score indicating a higher level of emotional distress. It uses a 5-point Likert scale ranging from: “No problem = 0”, “Small problem = 1”, “Moderate problem = 2”, “Almost a serious problem = 3”, and “Serious problem = 4”. A total score of 0-100 was achieved by summing the 0–4 responses given in the 20 PAID items and multiplying this sum by 1.25. The scale run time was 5–10 minutes.

### Statistical analysis

We described participants’ characteristics as mean and standard-deviation (quantitative data) or as an absolute number and percentage (qualitative data); and we calculated internal consistency using Cronbach's alpha, considering the variation between 0.70 and 0.95 as adequate values.^15^

We assessed reliability using a test-retest model with a 7-day interval between assessments. The intra-class correlation coefficient (ICC), 95% confidence interval (CI), standard error of measurement (SEM), and minimum detectable difference (MDD) were used to assess the reliability of the HASMID-10 total score. We considered an ICC value > 0.75 as adequate.^16^

Data normality was verified using the Kolmogorov-Smirnov test. We determined the validity of the construct using Spearman's correlation coefficient (rho), looking for a negative correlation between HASMID-10 and PAID, and hypothesized that the correlation magnitudes were less than 0.30.^13^

In addition, we evaluated floor and ceiling effects, which occurred when a number of study participants (more than 15%) reached the minimum or maximum value of the questionnaire's total score, indicating a problem when assessing the instrument's responsiveness. All statistical analyses were performed using SPSS statistical software (version 17.0; Chicago, Illinois, United States) with a significance level of 5%.

## RESULTS

During the translation phase, the Brazilian version of the HASMID-10 underwent one cross-cultural adaptation: in item 4, the term “going hypo” was adapted to “having a hypoglycemic crisis.” Thus, the pre-final version of the HASMID-10 was administered to 30 diabetic respondents with the understanding that all 10 items of the questionnaire were completely understood.

One hundred and twenty-nine participants were initially recruited for this study. From this sample, 13 respondents who completed the online form were excluded because they were below 18 years, leaving a final sample of 116 participants, most of whom were women, overweight, non-practitioners of physical activity, and non-smokers (Table 1).

**Table 1:** Descriptive analysis of personal and clinical characteristics (n = 116)

Variables	Mean (SD) or number (%)
Age (years)	53.97 (16.78)
Sex (female)	77 (66.4%)
Body mass (kg)	70.76 (19.35)
Stature (m)	1.60 (0.08)
Body mass index (kg/m^2^)	27.23 (6.40)
Physical activity (no)	94 (81%)
Smoke (no)	97 (83.6%)
Time of diabetes (years)	9.93 (8.63)
Problems areas in diabetes scale (score)	62.48 (27.49)
Health and self-management in diabetes questionnaire (score)	15.02 (5.89)

SD = standard deviation.

Regarding construct validity, as there is no instrument in Brazilian Portuguese that assesses the same construct as the HASMID-10, we used the PAID and observed significant correlations with a weak correlation magnitude (P = 0.006; rho = −0.256).

Thirty participants answered the HASMID-10 at two time points to analyze test-retest reliability (Table 2), and adequate reliability (ICC = 0.780) and internal consistency (Cronbach's alpha = 0.796) were observed. We observed that no participant obtained a minimum score, and two (1.7%) participants obtained a maximum score on the HASMID-10 (i.e., ceiling and floor effects were not observed).

**Table 2:** Reliability and internal consistency of the health and self-management in diabetes questionnaire (HASMID-10)

Test	Retest	ICC (CI 95%)	SEM	SEM (%)	MDD	MDD (%)	Cronbach's α
16.16 (3.02)	16.16 (3.00)	0.798 (0.741, 0.41)	1.40	8.64	3.87	23.94	0.796

CI = confidence interval; ICC = intraclass correlation coefficient; SEM = standard error of measurement; MDD = minimum detectable difference.

## DISCUSSION

First, we confirmed our hypothesis that the negative correlation between HASMID-10 (higher scores indicating better quality of life) and PAID (higher scores indicating worse emotional distress) had a weak magnitude. As such, by observing adequate values of reliability, internal consistency, and the absence of ceiling and floor effects, we recommended the applicability of the HASMID-10 for four reasons (described below).

First, the construct validity performed through correlation (P = 0.006; rho = −0.256) between a previously validated instrument (PAID)^14^ and an instrument of interest (HASMID-10)^11^ for validation in the same population (Brazilian) supports the applicability (clinical and scientific) of the instrument because construct validity makes it possible to determine whether the questionnaire of interest (HASMID-10) has the ability to measure the construct of interest (in this case, yes).^13^

Second, when confirming adequate reliability values, as in our study (ICC = 0.780), we are sure that the instrument measures what it actually proposes to measure.^13^ Besides, this measurement allows us to observe the values of the standard error of measurement (1.40) and minimum detectable difference (3.87), indicating what happened between the first and next clinical investigations while monitoring diabetes prognosis.^17^

Third, the instrument's internal consistency showed the interrelationship between the questionnaire's items^13^. This consistency (Cronbach's α = 0.796) ensures that an item helps to understand the other items and that the set of items will show (via patients’ self-report) the patients’ clinical condition.^18^ This is the first HASMID-10 validation study for another language (in this case, Brazilian Portuguese), although this is a strength of our study, it prevents internal consistency comparison with other publications.

Fourth, the absence of ceiling and floor effects (as in our study) ensures that the instrument applies to most patients who will be evaluated.^13^ Diabetes patients have systemic and wavering symptoms^19^ (e.g., sometimes serious symptoms, sometimes mild symptoms),^20–22^ making it difficult to evaluate the patient. To avoid this, it is necessary to evaluate a sample with average symptoms (i.e., values below maximum levels and above minimum levels)—as in our study.

Finally, our study has two limitations. First, most of the sample was female, and we recommend the reproducibility of this study for a balanced sample (male and female). Second, unfortunately, there are no other studies that have validated this instrument for other languages; thus, our discussion is limited, and we recommend adapting this instrument to other languages (cultures, countries).

## CONCLUSION

The HASMID-10 has adequate measurement properties and can be used in the Brazilian population. We recommend its use in both clinical practice and research.
